# Leisure-time versus full-day energy expenditure: a cross-sectional study of sedentarism in a Portuguese urban population

**DOI:** 10.1186/1471-2458-5-16

**Published:** 2005-02-15

**Authors:** Diane L Gal, Ana-Cristina Santos, Henrique Barros

**Affiliations:** 1 Department of Hygiene and Epidemiology, University of Porto Medical School, Porto, Portugal

## Abstract

**Background:**

Low physical activity is known to be a potential risk factor for cardiovascular disease. With high prevalence of cardiovascular diseases in the Portuguese urban population, little is known about how sedentary this population is and what factors are associated to sedentary lifestyles. This study's objective was to examine sedentary lifestyles and their determinants through a cross-sectional study.

**Methods:**

2134 adults (18 years and older) were interviewed using a standard questionnaire, comprising of social, behavioural and clinical information. Time spent in a variety of activities per day, including: work, household chores, sports, sedentary leisure time and sleep, were self-reported. Energy expenditure was estimated based on the related metabolic equivalent (MET) and time spent in each activity (min/day). Those with less than 10% of energy expenditure at a moderate intensity of 4 METs or higher were categorised as sedentary. The proportion of sedentary people and 95% Confidence Intervals (CI) were calculated, and the magnitude of associations, between sedentary lifestyles and the population characteristics, were computed as age-adjusted odds ratios using logistic regression.

**Results:**

Sedentarism in both genders during leisure time is high at 84%, however in full day energy expenditure, which includes physical activity at work, sleeping hours and household chores, 79% of males and 86% of females are found to be sedentary. In leisure-time only, increased age is associated with higher odds of being sedentary in both genders, as well as in women with increased BMI. In comparison, in full-day energy expenditure, sedentarism is more likely to occur in those with higher levels of education and in white-collar workers.

**Conclusions:**

A high prevalence of sedentarism is found in the study participants when measuring leisure-time and full-day energy expenditure. The Portuguese population may therefore benefit from additional promotion of physical activity.

## Background

Physical activity has been defined by World Health Organization [1] as comprising of all movements in everyday life, including work, recreation, and sports activities and has been categorised in levels of intensity from light to moderate to vigorous. Health benefits, including decreasing risk of coronary heart disease [2-5] have mostly been associated with moderate to vigorous activities [6]. Practicing 30 minutes of moderate physical activity at least five days a week is widely promoted to achieve health benefits and is also felt to be an achievable lifestyle change for sedentary adults [1].

Physical activity tends to be associated with lower cardiovascular morbidity and mortality and an overall improved quality of life. Some studies [6-9] have defined or examined sedentary lifestyles and associated factors at the population level, however few have approached the subject in the context of Southern Europe. This may be due in part to the fact that it is difficult to classify a person as sedentary since no universally accepted classification is currently available. Previous studies have used a simple question or an evaluation of whether adults perform at least 30 minutes of moderate activity five times a week [10], or did not take part in leisure time physical activity at all [4,11], to classify participants as inactive or sendentary.

Portugal has the highest stroke mortality rates in Western Europe and cardiovascular diseases cause approximately 40% of deaths [12,13]. The current study's objective was to examine sedentarism in leisure time and throughout a full day in a Portuguese urban population and to cross-sectionally assess the associations between sedentarism and demographic, social, behavioural, clinical and anthropometric factors.

## Methods

Data was obtained as part of an ongoing cross-sectional health survey of adults living in the city of Porto, Portugal. Random digit dialling was used to select a single person over 17 years old from each household, without allowing for substitution of refusals. A participation rate of 70% was achieved [14]. Using a structured questionnaire, trained interviewers collected data from 2134 adults on demographic, personal and family medical history, and behavioural characteristics (physical activity, smoking, alcohol intake and diet) [15]. Sixty-seven participants who scored less than 24 on the Folstein mini-mental state examination [16] were considered probably unable to provide reliable information due to cognitive impairment and were excluded from the analysis. An additional 16 participants who did not fit the survey criteria (did not live in Porto or had severe disabilities and diseases) were also excluded. As well, participants missing relevant data were not included in the study analyses, leaving 2004 participants (1226 women; 778 men) in the analysis.

As reported in an earlier publication [17], education was recorded as completed years of schooling and divided into three broad categories: less than 5, 5–11, and more than 11 years. Body weight was measured to the nearest 0.1 kg using a digital scale, and height was measured to the nearest centimetre in the standing position using a wall standiometer. Body mass index (BMI) was calculated as weight in kilograms divided by square height in meters. The distribution of BMI is reported by standard WHO categories and nomenclature [18]: underweight to normal (<25.0 kg/m2), overweight (25.0–29.9 kg/m2), and obese (> = 30 kg/m2). The number of self-reported medical visits occurring in the last 12 months was grouped based on tertiles (0–1, 2–3, >3). Current occupation was self-reported and divided into the three usual categories of white collar, blue-collar and retired or unemployed. White collar work included all non-manual and superior professionals such as teachers, health professionals, secretaries etc. Blue-collar work included all manual professionals including agriculturers, taxi drivers, cooks, factory workers and sewers. Women who stated that they performed domestic work in their own home and had no other employment were classified as unemployed. Each participant was also asked about chronic diseases requiring continued medical care. Energy intake was estimated based on a semi-quantitative food frequency questionnaire validated for the Portuguese population and results were presented in tertiles, separately for each gender. Alcohol intake was self-reported and classified into three categories: current drinkers (daily alcohol intake), ex-drinkers (no alcohol for more than 6 months), and never or occasional drinkers. Smoking was self-reported and classified based on the WHO categories [19]: current smoker included both daily and occasional smokers, ex-smokers were those who had not smoked a cigarette in the last 6 months, and non-smokers were those who never smoked at all.

Participants completed a physical activity questionnaire designed to estimate usual individual daily energy expenditure, focused on the activity in the past year. Time spent in a variety of activities per day, including: work, transport to or from work, household chores, sports, sedentary leisure time and sleep, was self-reported and activity intensity categorised as very light, light, moderate and heavy with a corresponding average of 1.5, 2.5, 5.0 and 7.0 METs respectively, where one MET is equal to the energy expended at the basal metabolic rate or at rest [20]. Due to the manner in which these questions were presented during the face-to-face interviews, a large variation resulted in the number of hours reported per day (Average = 19 hours/day (minimum = 6.5 to maximum = 32.5 hours) with 46 (2.3%) participants reporting activity resulting in more than 24 hours per day). Energy expenditure was estimated by multiplying the related metabolic equivalent (MET) to the self-reported time spent in each activity (min/day). Participants with less than 10% of daily energy expenditure at a moderate or high intensity level (>4 METs) during leisure-time or throughout the day were categorised as sedentary, the remaining being considered active [21]. Proportions of sedentary individuals and 95% CI were calculated for both leisure-time and full-day energy expenditure, the latter including energy expended at work, during sleep and in household chores. The magnitude of associations, between sedentary lifestyles and the factors studied, were computed as age-adjusted odds ratios using logistic regression. In the analyses of the percentages of sedentarism and its associations with leisure-time energy expenditure, 15 people were excluded since they did not report any leisure-time activities. Analyses were conducted using Stata 7.0.

## Results

In the exploration of the population studied it was found that a significant difference between men and women was noted in most baseline characteristics other than in the age distribution and the hours of sleep (mean hours of sleep per night equalled 8; 95%CI 7.9–8.1). A higher proportion of household work was undertaken by women (95.5% versus 55.5% of men), a higher proportion of men were married (84.3% versus 61.3% of women), 68.3% of women and 55.7% of men reported no chronic disease. 31% of all participants worked between 20 and 40 hours a week, with a higher percentage of men working greater than 40 hours (31.1% versus 15.2% of women). It is also worthy to note that a high percentage of both genders reported not undertaking regular leisure-time sports and exercise (69.3% of women and 58.9% of men). All subsequent analyses were performed separately for males and females.

Overall sedentary lifestyle percentages (Table 1 and 2) are high, 83.7% (95%CI: 80.9–86.2) for males and 84.4% (95%CI: 82.2–86.3) for females during leisure time. A lower percentage was found for males with 78.8% (95%CI: 75.7–81.6) when the full day energy expenditure is calculated including physical activity at work, hours of sleep and household chores. The full-day sedentary lifestyle percentage for women, however, increased slightly to 86.1% (95%CI: 84.0–88.0).

**Table 1:** Sedentarism in the Female Population

	**Characteristics**	Leisure-time Energy Expenditure *		Full-day Energy Expenditure **	
	
	N (%)	% (95% CI)	OR^† ^(95% CI)	% (95% CI)	OR^† ^(95% CI)
	1226 (61.2)	84.4 (82.2–86.3)		86.1 (84.0–88.0)	
Age (years)					
18–29	88 (7.2)	68.2 (57.4–77.7)		75.0 (64.6–83.6)	
30–39	125 (10.2)	71.4 (62.4–79.3)	1.2 (0.6–2.1)	77.6 (69.3–84.6)	1.2 (0.6–2.2)
40–49	294 (24.0)	84.6 (80.0–88.6)	2.6 (1.5–4.5)	83.3 (78.6–87.4)	1.7 (0.9–3.0)
50–59	311 (25.4)	85.1 (80.5–88.8)	2.7 (1.5–4.6)	85.2 (80.7–88.9)	1.9 (1.1–3.4)
60–69	238 (19.4)	91.5 (87.2–94.7)	5.0 (2.7–9.6)	90.8 (86.3–94.1)	3.3 (1.7–6.3)
70+	170 (13.9)	90.0 (84.5–94.1)	4.2 (2.1–8.2)	98.2 (94.9–99.6)	18.6 (5.4–64.1)
Marital Status					
Married	751 (61.3)	85.3 (82.5–87.7)		86.4 (83.7–88.7)	
Not married	475 (38.7)	82.9 (79.1–86.1)	0.8 (0.6–1.2)	85.7 (82.1–88.6)	0.8 (0.6–1.2)
Education (years)					
<5 years	530 (43.2)	93.7 (91.2–95.6)		87.0 (83.7–89.7)	
5–11 years	330 (26.9)	84.5 (80.0–88.1)	0.4 (0.2–0.6)	83.9 (79.4–87.6)	1.2 (0.8–1.8)
>11 years	366 (29.9)	70.6 (65.6–75.2)	0.2 (0.1–0.3)	86.9 (82.9–90.1)	2.2 (1.4–3.6)
BMI (kg/m2)					
<25	455 (37.1)	77.5 (73.2–81.2)		85.3 (81.6–88.3)	
25–30	452 (36.9)	85.1 (81.4–88.2)	1.3 (0.9–1.9)	85.2 (81.5–88.3)	0.7 (0.5–1.0)
>30	319 (26.0)	93.1 (89.6–95.5)	2.9 (1.8–4.9)	88.7 (84.6–91.9)	0.9 (0.6–1.4)
Physician visits in last year (n)					
0–1	422 (34.4)	84.4 (80.5–87.7)		84.4 (80.5–87.6)	
2–3 visits	381 (31.1)	79.1 (74.6–83.0)	0.6 (0.4–0.8)	84.8 (80.7–88.2)	0.8 (0.6–1.2)
>3	423 (34.5)	89.0 (85.6–91.8)	1.2 (0.8–1.8)	89.1 (85.7–91.9)	1.1 (0.7–1.7)
Occupation					
White collar worker	425 (34.7)	77.1 (72.7–81.0)		88.2 (84.7–91.1)	
Blue collar worker	193 (15.7)	92.1 (87.3–95.5)	3.0 (1.7–5.4)	61.7 (54.4–68.5)	0.2 (0.1–0.3)
Unemployed or retired	608 (49.6)	87.0 (84.0–89.5)	1.4 (0.9–2.1)	92.4 (90.0–94.4)	1.0 (0.6–1.6)
Energy Intake (kcal/day)					
<1800	400 (32.7)	83.3 (79.3–86.9)	1.0 (0.7–1.5)	86.8 (83.0–89.9)	1.0 (0.7–1.5)
1800–2300	461 (37.7)	82.7 (78.8–86.0)		85.7 (82.1–88.7)	
>2300	362 (29.6)	87.5 (83.6–90.7)	1.8 (1.2–2.7)	85.9 (81.9–89.3)	1.3 (0.9–1.9)
Alcohol Use					
Non/Occasional-drinkers	576 (47.0)	80.7 (77.2–83.8)		83.7 (80.3–86.6)	
Ex-drinkers	103 (8.4)	94.2 (87.8–97.8)	2.6 (1.1–6.2)	93.2 (86.5–97.2)	1.7 (0.8–4.0)
Drinkers	547 (44.6)	86.3 (83.1–89.0)	1.3 (0.9–1.3)	87.4 (84.2–90.0)	1.2 (0.9–1.8)
Tobacco Use					
Non-smokers	893 (72.8)	84.9 (82.3–87.2)		86.6 (84.1–88.7)	
Ex-smokers	119 (9.7)	81.7 (73.5–88.3)	0.9 (0.6–1.6)	89.1 (82.0–94.1)	1.6 (0.9–3.0)
Smokers	214 (17.5)	83.6 (77.9–88.3)	1.5 (0.9–2.3)	82.7 (77.0–87.5)	1.2 (0.8–1.8)

* Leisure time energy expenditure encompasses the energy expended in all leisure activities (not including sleep, work and household chores) where being sedentary is defined as spending less than 10% of their time in activities requiring ≥ 4 metabolic equivalents (MET).

** Full-day energy expenditure encompasses the energy expended in all activities in a day where being sedentary is defined as above.

† Age-adjusted Odds ratios

**Table 2:** Sedentarism in the Male Population

	**Characteristics**	Leisure-time Energy Expenditure *		Full-day Energy Expenditure **	
	
	N (%)	% (95% CI)	OR^† ^(95% CI)	% (95% CI)	OR^† ^(95% CI)
	778 (38.8)	83.7 (80.9–86.2)		78.8 (75.7–81.6)	
Age (years)					
18–29	49 (6.3)	57.1 (42.2–71.2)		69.4 (54.6–81.7)	
30–39	68 (8.7)	74.6 (62.5–84.5)	2.2 (1.0–4.9)	73.5 (61.4–83.5)	1.2 (0.5–2.8)
40–49	173 (22.2)	79.2 (72.4–85.0)	2.9 (1.5–5.6)	78.0 (71.1–84.0)	1.6 (0.8–3.2)
50–59	180 (23.1)	85.6 (79.6–90.3)	4.4 (2.2–9.0)	76.7 (69.8–82.6)	1.4 (0.7–2.9)
60–69	174 (22.4)	89.5 (84.0–93.7)	6.4 (3.0–13.5)	80.5 (73.8–86.1)	1.8 (0.9–3.7)
70+	134 (17.2)	94.0 (88.5–97.4)	11.7 (4.7–29.2)	86.6 (79.6–91.8)	2.8 (1.3–6.2)
Marital Status					
Married	656 (84.3)	85.1 (82.1–87.7)		78.7 (75.3–81.7)	
Not married	122 (15.7)	76.2 (67.7–83.5)	1.1 (0.6–2.0)	79.5 (71.3–86.3)	1.4 (0.8–2.5)
Education (years)					
<5 years	262 (33.7)	93.4 (89.7–96.1)		74.8 (69.1–79.9)	
5–11 years	282 (36.2)	84.0 (79.2–88.1)	0.5 (0.3–0.9)	78.7 (73.5–83.4)	1.6 (1.0–2.4)
>11 years	234 (30.1)	72.5 (66.3–78.2)	0.3 (0.2–0.5)	83.3 (77.9–87.9)	2.5 (1.5–4.2)
BMI (kg/m2)					
<25	279 (35.9)	79.9 (74.7–84.4)		77.4 (72.1–82.2)	
25–30	375 (48.2)	84.9 (80.8–88.3)	1.2 (0.8–1.9)	78.7 (74.1–82.6)	1.1 (0.7–1.6)
>30	124 (15.9)	88.7 (81.8–93.7)	1.8 (0.9–3.4)	82.3 (74.4–88.5)	1.3 (0.8–2.3)
Physician visits in last year (n)					
0–1	354 (45.5)	80.9 (76.3–84.8)		76.0 (71.1–80.3)	
2–3 visits	236 (30.3)	84.7 (79.4–89.0)	1.1 (0.7–1.7)	79.2 (73.5–84.2)	1.1 (0.7–1.7)
>3	188 (24.2)	87.8 (82.2–92.1)	1.1 (0.6–1.9)	83.5 (77.4–88.5)	1.4 (0.9–2.3)
Occupation					
White collar worker	336 (43.2)	83.1 (79.5–86.2)		85.4 (82.0–88.3)	
Blue collar worker	133 (17.1)	86.6 (81.8–90.6)	1.6 (0.9–2.9)	65.6 (59.4–71.5)	0.2 (0.1–0.3)
Unemployed or retired	309 (39.7)	53.8 (25.1–80.8)	0.8 (0.5–1.4)	69.2 (38.6–90.9)	0.6 (0.4–1.0)
Energy Intake (kcal/day)					
<2300	261 (33.7)	88.1 (83.6–91.8)	1.1 (0.7–1.8)	86.6 (81.8–90.5)	1.6 (1.0–2.5)
2300–2900	283 (36.5)	84.3 (79.6–88.4)		79.5 (74.3–84.1)	
>2900	231 (29.8)	77.7 (71.8–82.9)	0.8 (0.5–1.3)	68.8 (62.4–74.7)	0.6 (0.4–0.9)
Alcohol Use					
Non/Occasional drinkers	87 (11.2)	80.2 (70.2–88.0)		82.8 (73.2–90.0)	
Ex-drinkers	48 (6.2)	83.3 (69.8–92.5)	0.7 (0.2–1.8)	79.2 (65.0–89.5)	0.6 (0.2–1.5)
Drinkers	643 (82.7)	84.2 (81.1–86.9)	1.0 (0.5–1.8)	78.2 (74.8–81.3)	0.7 (0.4–1.2)
Tobacco Use					
Non-smokers	218 (28.0)	82.6 (76.9–87.4)		78.9 (72.9–84.1)	
Ex-smokers	296 (38.1)	86.1 (81.6–89.8)	1.0 (0.6–1.6)	77.0 (71.8–81.7)	0.8 (0.5–1.3)
Smokers	264 (33.9)	82.1 (76.9–86.5)	1.2 (0.7–1.9)	80.7 (75.4–85.3)	1.3 (0.8–2.0)

* Leisure time energy expenditure encompasses the energy expended in all leisure activities (not including sleep, work and household chores) where being sedentary is defined as spending less than 10% of their time in activities requiring ≥ 4 metabolic equivalents (MET).

** Full-day energy expenditure encompasses the energy expended in all activities in a day where being sedentary is defined as above.

^†^Age-adjusted Odds ratios

Few differences were found in the level of sedentarism in adults when considering differences in population characteristics. Younger participants tend to have lower percentages of sedentarism compared to older participants. In the leisure-time only estimation unmarried men (76.2%; 95%CI: 67.7–83.5) and female white-collar workers (77.1%; 95%CI: 72.7–81.0) tend to be more active. Women and men tend to be more active in leisure-time with increasing years of education, changing from 94% sedentarism in those with less than five years of education to 84% for those with five to eleven years of education and 72.5% for men and 70.6% for women with greater than 11 years of schooling. However, when the full-day energy expenditure is used as the estimate, these differences are no longer found and the trends with education and occupation are reversed. The lowest levels of sedentarism are found in males (74.8%; 95%CI: 69.1–79.9) with less education, and men who have a high energy intake (68%; 62.4–74.7) as well as, both male (65.6%; 95%CI: 59.4–71.5) and female (61.7%; 95%CI: 54.4–68.5) blue-collar workers.

When further examining the results of the age-adjusted associations between the population characteristics and sedentarism it is found that few factors were associated with an increased proportion of sedentarism. Marital status, physician visits in the last year and tobacco consumption, once adjusted for age, were not associated with differences in energy expenditure and sedentarism. In the leisure-time only estimation, increased age was associated with higher odds of being sedentary in both males and females. Sedentarism increased with increased BMI in women (BMI 25–30 = OR 1.3 95% CI: 0.9–1.9 ; BMI >30 = OR 2.9 95%CI:1.8–4.9), as well as in women with a high energy intake (>2300 Kcal/day = OR 1.8 95%CI:1.2–2.7). Sedentarism also increased in males who were ex-drinkers when compared to non or occasional drinkers (OR 2.6 95%CI: 1.1–6.2). Following what was noted earlier, higher levels of education were associated with higher levels of activity in leisure-time in both males (5–11 years education = OR 0.5 95%CI: 0.3–0.9; >11 years education = OR 0.3 95%CI:0.2–0.5) and females (OR 0.4 95%CI: 0.2–0.6 and OR 0.2 95%CI: 0.1–0.3, respectively) and blue-collar workers were more likely to be sedentary (males: OR 1.6 95%CI: 0.9–2.9; females: OR 3.0 95%CI: 1.7–5.4).

In comparison, in the full-day energy expenditure estimation, increased odds in age were not as strong for men and evidence of an association was not apparent with increased BMI or calorie intake for women. However, the reverse association was identified where those with higher amounts of education tended to be more sedentary in both males (5–11 years education = OR 1.6 95%CI: 1.0–2.4; >11 years education = OR 2.5 95%CI:1.5–4.2) and females (OR 1.2 95%CI: 0.8–1.8 and OR 2.2 95%CI: 1.4–3.6, respectively) and blue-collar workers were found to be significantly less sedentary (OR 0.2 95%CI:0.1–0.3). Relationships with energy intake also were identified in men with those consuming less than 2300 Kcal/day on average having a higher odds of being sedentary (OR 1.6 95%CI: 1.0–2.5) and those consuming greater than 2900 Kcal/day having a lower odds of being sedentary (OR 0.6 95%CI:0.4–0.9).

## Discussion

Our results highlight the primarily sedentary nature of this adult urban population, with 70% of women and 60% of men not undertaking any regular physical activity or sports during leisure time. Similar studies, which have only evaluated leisure time physical activity, have identified comparable levels of sedentarism, as well as associations between sedentarism and certain population factors. In a European Union study [22] conducted in 1997, it was reported that the Portuguese population was one of the most sedentary among the 15 countries studied, with 85.2% of men and 90.0% of women being classified as sedentary compared to 83.7% of men and 84.4% of women in this study. It would be expected that, since the sampling in the study was meant to be representative of the whole country, a greater difference in the overall levels of sedentarism would be found mainly due to the differences in the levels and types of activities undertaken by rural and city dwellers. The small sample number in the European study (1007 participants) may also not capture the full extent of activities undertaken by the population in general. Similar associations were also noted for leisure-time energy expenditure, where the prevalence of sedentarism was higher with age and higher in the less educated in the European and in a Swiss [23] study. Other associations found in the European study, that obese individuals had higher prevalence of sedentarism was only found to be true in women in our study and no association was found between sedentarism and current smoking as identified in the European study. Lower levels of physical activity have also been associated with those who were female, older and with lower socio-economic status in a New Zealand study [10]. The differences between other studies results and ours may be due to true differences between the study sample baseline characteristics, or possible due to the study methods utilised.

The questionnaire, which was used to collect data on physical activity, was developed according to the European Prospective Investigation into Cancer and Nutrition study questionnaire, which showed acceptable repeatability and validity [24]. Formal validation of the questionnaire was undertaken using four seven days records (data not published). Participant recall may limit accurate capturing, through the questionnaire, of time and intensity spent undertaking various activities [25] during an average day or week in the last year. Although a variation in the hours of activities reported in a day was found, the percentage of participants reporting over 24 hours of activity was small (2.2%) and would not substantially affect the results of the study. As well, the description of types of activities provided in the questionnaire allow for METs to be estimated based on the Compendium of physical activity[20]. Variation is also present between studies in the categorisation of metabolic equivalents for activities with moderate intensity, with the US Surgeon General reporting moderate intensity exercise as being equal to 3–5 times the basal metabolic rate [26], while other studies use a cut off for moderate activity being more than 4, [2,21,22] or even greater than 5 METs [8]. Thirty minutes of activity at 4 METs, in an adult with 75 kg, will lead to an approximate energy expenditure of 150 Kcal per day or 1050 Kcal per week, which is a minimum level of moderate intensity daily activity recommended in the US Surgeon General report [26]. As energy expenditure varies from person to person, previous studies [21,22] have measured energy expenditure and have defined someone as being sedentary if they expend less than 10% of their daily energy in the performance of moderate-intensity activities (at least 4 times the basal metabolism rate) and therefore, on average, expend less than the recommended150 Kcal per day.

The above mentioned studies only recorded and based results on leisure-time energy expenditure, excluding the potential input of physical activity that is undertaken at work or on household tasks. As presented in the results of this study, the inclusion of work-time energy expenditure shows that those less educated and those with manual occupations are less sedentary, which reverses the association seen in the leisure-time only estimation. The differences between the associations of the two separate measurements, leisure-time energy expenditure versus full-day energy expenditure, therefore demonstrate the potential for work-related and household-related physical activity to significantly affect the proportion of sedentarism, and the associations between sedentarism and the factors studied. Efforts, therefore, need to be made to include all components of daily physical activity and energy expenditure and to study the effects of this energy expenditure as a whole, on cardiovascular disease and other health benefits of moderate and high-intensity energy expenditure, which has also been highlighted by Salmon et al [27]. However, the different psychosocial aspects expectedly associated with the decision of engaging in leisure time physical activity or related to hard work as part of occupational tasks might result in different effects on health for the same amount of energy expenditure.

The European Society of Cardiology has outlined, in a recent position paper, the need for physical activity to be prescribed in primary and secondary prevention and to implement successful strategies to reduce cardiovascular risks [28]. It has been observed for centuries that physical activity maintains and improves health and well-being, however health-systems have done little to promote and support appropriate levels of physical activity, especially in groups with elevated cardiovascular risk [29]. The lack of knowledge of the determinants of, and health problems related to, sedentarism and of the best interventions for behavioural change and long-term adherence to physical activity may play a part in low prescription of physical activity. Interventions to decrease sedentarism through primary health care [11] and in workplace settings [30] have had positive results, however all interventions may not affect change, such as was found with a population-wide print-media intervention [31]. Lessons can be learned from these interventions, and appropriate public health interventions prepared, in order to reduce the high levels of sedentarism, which acts as a main factor in high cardiovascular risk.

## Conclusions

The urban Portuguese population has a very high prevalence of reported sedentarism potentially contributing to the high levels of cardiovascular disease in the country. Caution, however needs to be taken in the classification of individuals as sedentary when considering leisure-time versus full-day energy expenditure, as work and household-related activities can account for a large portion of the energy spent. Including these measures may also affect the overall associations found between sedentarism and the population characteristics.

## Abbreviations

BMI Body Mass Index

MET Metabolic Equivalent

RMR Resting Metabolic Rate

WHO World Health Organisation

## Competing interests

The author(s) declare that they have no competing interests.

## Authors' contributions

DG designed the study, performed the statistical analysis and drafted the manuscript. ACS participated in the design of the study and in the interpretation of the results. HB conceived the study, and participated in its design and coordination. All authors read and approved the final manuscript.

## Pre-publication history

The pre-publication history for this paper can be accessed here:


